# The *situ* preparation of silica nanoparticles on the surface of functionalized graphene nanoplatelets

**DOI:** 10.1186/1556-276X-9-172

**Published:** 2014-04-09

**Authors:** Jiani Li, Kejing Yu, Kun Qian, Haijian Cao, Xuefeng Lu, Jie Sun

**Affiliations:** 1 Key laboratory of Science and Technology of Eco-Textile, Ministry of Education, Jiangnan University, Wuxi, Jiangsu 214122, People's Republic of China

**Keywords:** Graphene, SiO_2_ particles, Hybrid material, *Situ* preparation, Controllability

## Abstract

A method for *situ* preparing a hybrid material consisting of silica nanoparticles (SiO_2_) attached onto the surface of functionalized graphene nanoplatelets (f-GNPs) is proposed. Firstly, polyacrylic acid (PAA) was grafted to the surface of f-GNPs to increase reacting sites, and then 3-aminopropyltriethoxysilane (APTES) KH550 reacted with abovementioned product PAA-GNPs to obtain siloxane-GNPs, thus providing reaction sites for the growth of SiO_2_ on the surface of GNPs. Finally, the SiO_2_/graphene nanoplatelets (SiO_2_/GNPs) hybrid material is obtained through introducing siloxane-GNPs into a solution of tetraethyl orthosilicate, ammonia and ethanol for hours' reaction. The results from Fourier transform infrared spectroscopy (FTIR) showed that SiO_2_ particles have *situ* grown on the surface of GNPs through chemical bonds as Si-O-Si. And the nanostructure of hybrid materials was characterized by scanning electron microscopy (SEM) and transmission electron microscopy (TEM). All the images indicated that SiO_2_ particles with similar sizes were grafted on the surface of graphene nanoplatelets successfully. And TEM images also showed the whole growth process of SiO_2_ particles on the surface of graphene as time grows. Moreover, TGA traces suggested the SiO_2_/GNPs hybrid material had stable thermal stability. And at 900°C, the residual weight fraction of polymer on siloxane-GNPs was about 94.2% and that of SiO_2_ particles on hybrid materials was about 75.0%. However, the result of Raman spectroscopy showed that carbon atoms of graphene nanoplatelets became much more disordered, due to the destroyed carbon domains during the process of chemical drafting. Through orthogonal experiments, hybrid materials with various sizes of SiO_2_ particles were prepared, thus achieving the particle sizes controllable. And the factors’ level of significance is as follows: the quantity of ammonia > the quantity of tetraethyl orthosilicate (TEOS) > the reaction time.

## Background

Graphene, a single layer carbon material in a close arrangement of honeycomb two-dimensional lattice [1], has remarkable properties, such as Young's modulus, fracture strength, specific surface area and so on [2-4]. Significantly, graphene is a promising building block material for composites because of its large surface area. Furthermore, decoration of the graphene nanosheets with organic/inorganic materials can bring about an important kind of graphene-based composites [5-10]. However, the two-dimensional structure and huge specific surface area of graphene nanoplatelets made it easy to aggregate, which limited its application [11]. Thus it is necessary to overcome graphene's extreme hydrophobicity which leads to aggregation in polar liquids [12,13]. Researches indicated that the modification of graphene nanoplatelets is arguably the most versatile and easily scalable method [14]. Meaningfully, the decoration of nanomaterials onto graphene nanosheets is helpful to overcome the aggregation of individual graphene nanosheets and nanomaterials themselves [15]. In recent years, researchers have shown an increasing interest in graphene-based composites [16,17] in which graphene sheets are used as a wild phase to enhance mechanical properties [18]. Among all these materials, hybrid materials based on GNPs and silica nanoparticles have attracted significant scientific interest because of their remarkable properties that do not exist in the individual components [19-22]. Due to the synergistic effect, graphene nanoplatelets/SiO_2_ hybrid materials have superior properties compared with bare graphene nanoplatelets and SiO_2_ particles [23]. Considering the outstanding properties of graphene nanoplatelets and SiO_2_, graphene/silica composite would be one of the greatly popular and interest topics in the field of nanomaterial and nanotechnology [24]. And this kind of composite materials have been explored as adsorbents [25,26], catalysts [27], and fillers into resin for composites along with an excellent application potential [28,29].

Hao [11] et al. prepared SiO_2_/graphene composite for highly selective adsorption of Pb (II) ion through a simple two-step reaction, including the preparation of SiO_2_/graphene oxide and the reduction of graphene oxide (GO). Zhou [24] et al. used a one-pot hydrothermal synthesis to obtain a mesoporous SiO_2_-graphene hybrid from tetraethyl orthosilicate and graphene oxide without any surfactant. Lu [30] et al. reported on the preparation of well-defined SiO_2_-coated graphene oxide (GO) nanosheets (SiO_2_/GO) without prior GO functionalization by combining sonication with solgel technique. And then, the product is decorated with Ag nanoparticles for H_2_O_2_ and glucose detection. However, all these abovementioned method did not have the advantage of controlling the size of SiO_2_. Accordingly, the development of new preparation strategy overcoming the shortcoming is highly desired.

**Figure 1:**
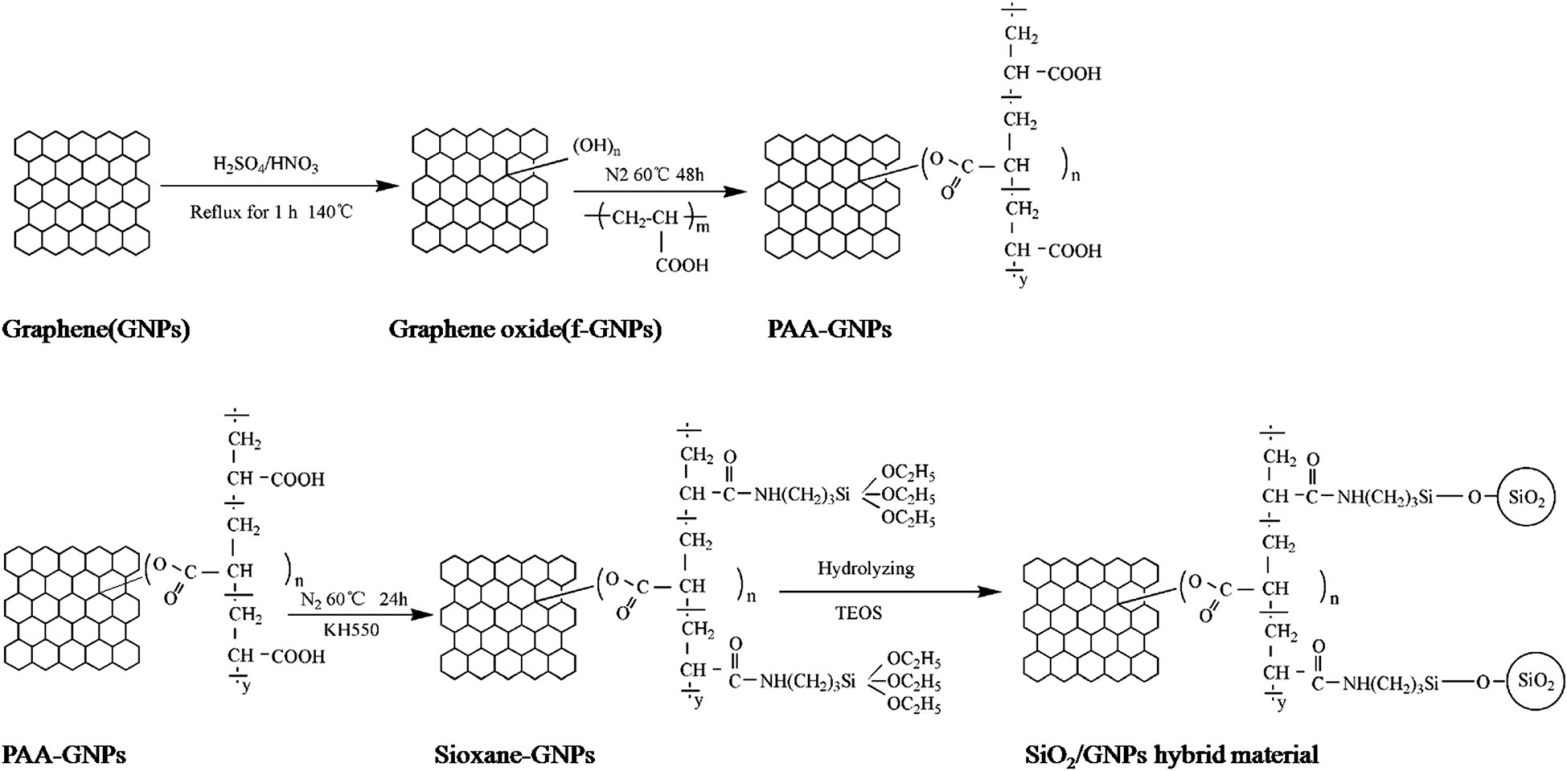
The schematic diagram of the reaction.

In our previous work, we introduced an easy and facial methodology to prepare functionalized graphene nanoplatelets (f-GNPs/SiO_2_) hybrid materials, using polyacryloyl chloride (PACl) as the bridge to connect graphene platelets and SiO_2_ particles. We have also introduced a facile approach to prepare multiwalled carbon nanotubes/graphene nanoplatelets hybrid materials. In this paper, we proposed a strategy to *situ* prepare SiO_2_ particles with similar sizes onto the surface of graphene nanosheets. The schematic diagram of reaction is illustrated in Figure 1. At first step, graphene nanosheet was acid treated by H_2_SO_4_/HNO_3_ (30 ml/30 ml) at 140°C for 1 h. Then, polyacrylic acid (PAA) was grafted onto the surface of f-GNPs through chemical bond C-O. And KH550 reacted with above mention product PAA-GNPs through chemical bond C-C = O to obtain siloxane-GNPs. Finally, the SiO_2_/GNPs hybrid material is produced through introducing siloxane-GNPs into a solution of tetraethyl orthosilicate, ammonia and ethanol for hours’ reaction. This approach is easy to control and efficient. Meaningfully, the size of situ general silica nanoparticles could be readily controlled by adjusting the ammonia concentration in the aqueous solution and the reaction time. There are various factors that can affect the size of SiO_2_ particles [31]. In present work, through orthogonal experimental design [32], we discuss the impact of following three factors on the size of SiO_2_ particles: the quantity of tetraethyl orthosilicate (TEOS), the quantity of ammonia and the reaction time.

## Methods

### Experimental section

#### Materials

Graphene nanoplatelets (GNPs) (diameter, 1 to 20 μm; thickness, 5 to 15 nm) were purchased from Xiamen Kona Graphene Technology Co., Ltd. (Xiamen, China). PAA (PH: 1–2) was purchased from Tianjin Damao chemical reagent Co. Ltd. N,N-Dicyclohexyl carbodiimide (DCC) was purchased from Aladdin industrial corporation, Seattle, Washington D.C., USA. 3-Aminopropyltriethoxysilane (APTES) KH550 was purchased from Shanghai Yaohua Chemical Co. Ltd., Shanghai, China. H_2_SO_4_ (98%), HNO_3_ (65%), tetrahydrofuran (analytically pure), TEOS (AR), ammonia solution (AR), and ethanol (AR) were provided by Sinopharm Chemical Reagent Co. Ltd. (Shanghai, China).

### Oxidation of graphene nanoplatelets

GNPs (900 mg) were suspended and refluxed in a mixture of concentrated acid H_2_SO_4_/HNO_3_ (30 ml/30 ml) at 140°C for 1 h, followed by diluting with deionized water (3,000 ml). The acid-treated GNPs were retrieved and washed repeatedly with THF until pH = 7 and dried under vacuum. The product was denoted as f-GNPs.

### Grafting PAA onto f-GNPs

f-GNPs 50 mg, PAA 100 mg, DCC 100 mg, and THF 50 ml were mixed under dry nitrogen atmosphere and then stirred in a flask for 48 h at 60°C. The solid product was collected and washed repeatedly with THF until pH = 7 and dried under vacuum. The product was denoted as PAAGNPs.

### Reaction of PAA-GNPs and KH550

PAA-GNPs 100 mg, DCC 100 mg and THF 100 mg were mixed by sonication for 1 h. Then, the solution of KH550 was added dropwise into suspension at 60°C under nitrogen atmosphere. When completed, the reaction was kept at 60°C and vigorously stirred for 24 h. At last, the solid product was collected and washed repeatedly with THF until pH = 7 and dried under vacuum. The KH550 functionalized GNPs were denoted as siloxane-GNPs.

### Preparation of SiO_2_/GNPs hybrid material

Siloxane-GNPs (50 mg) were added into 10 ml deionized water and stirred for 24 h at room temperature to hydrolyze the alkoxysilane into Si-OH. Then, 0.6 g TEOS, 1.2 g ammonia solution, and 100 ml ethanol were added to the suspension and stirred for 8 h. Finally, the solid product was collected and washed repeatedly with THF until pH = 7 and dried under vacuum. In this process, the quantity of TEOS, the quantity of ammonia, and the time of reaction can be different. Thus, we can control the size of SiO_2_ particles.

### Orthogonal array experimental design

In the present study, the experiment was based on an orthogonal array experimental design where the following three factors were analyzed: the quantity of TEOS, the quantity of ammonia and the reaction time. These variables were identified to have large effects on the growth of SiO_2_ particles. So an orthogonal array of three factors and three levels was employed to assign the considered factors and levels as shown in Table 1. In principle, one column could be assigned to a factor. Here, the matrix denotes three factors, each with three levels (Table 2). Data analysis could be carried out through the range analysis.

**Table 1:** Levels of factor of orthogonal design

**Level**		**Factors**	
	**TEOS (g)**	**NH**_**3**_ **· H**_**2**_**O (g)**	**Time (h)**
1	0.3	0.6	4
2	0.6	1.2	6
3	0.9	1.8	8

**Table 2:** Orthogonal arrays for statistical experiment and results

**No.**	**Experiment conditions**					**Results**
	**Ethanol (ml)**	**Temperature (°C)**	**TEOS (g)**	**NH**_**3**_ **· H**_**2**_**O (g)**	**Time (h)**	**Average particle size (nm)**
1	100	30	0.3 (1)	0.6 (1)	4 (1)	50
2	100	30	0.3 (1)	1.2 (2)	6 (2)	120
3	100	30	0.3 (1)	1.8 (3)	8 (3)	140
4	100	30	0.6 (2)	0.6 (1)	6 (2)	100
5	100	30	0.6 (2)	1.2 (2)	8 (3)	240
6	100	30	0.6 (2)	1.8 (3)	4 (1)	170
7	100	30	0.9 (3)	0.6 (1)	8 (3)	130
8	100	30	0.9 (3)	1.2 (2)	4 (1)	160
9	100	30	0.9 (3)	1.8 (3)	6 (2)	280

### Characterizations

Fourier transform infrared spectrometer (FTIR, Nexus 670, Valencia, CA, USA) was used to detect the functional groups on the surface of f-GNPs and f-GNPs/SiO_2_ hybrid materials, which was measured as pellets with KBr. Raman spectroscopy (In Via laser confocal microscope, Renishaw, Wotton-under-Edge, UK) was employed to investigate the ordered or disordered crystal structures and assessing defects of samples, which was recorded using a spectrometer with 532 nm wavelength incident laser light. Thermal gravimetric analysis (TGA, SDTA851e) was used to evaluate the weight loss ratio of the products. The tests were conducted at a heating rate of 10°C/min from room temperature to 900°C under nitrogen. Scanning electron microscopy (SEM, HITACHI SU1510, Chiyoda-ku, Japan) was employed to observe the surface morphology of various products, whose accelerating voltage was 1.0 kV. Transmission electron microscopy (TEM, H-800-1) was employed to observe the microstructure of various products, whose accelerating voltage was 20 kV.

## Results and discussion

### Fourier transform infrared spectroscopy

**Figure 2:**
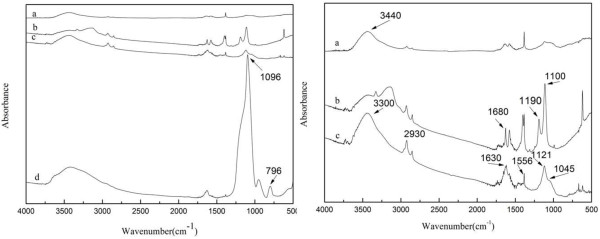
**FTIR spectra of (a) f-GNPs, (b) PAA-GNPs, (c) siloxane-GNPs, and (d) SiO**_**2**_**/GNPs hybrid material.**

**Figure 3:**
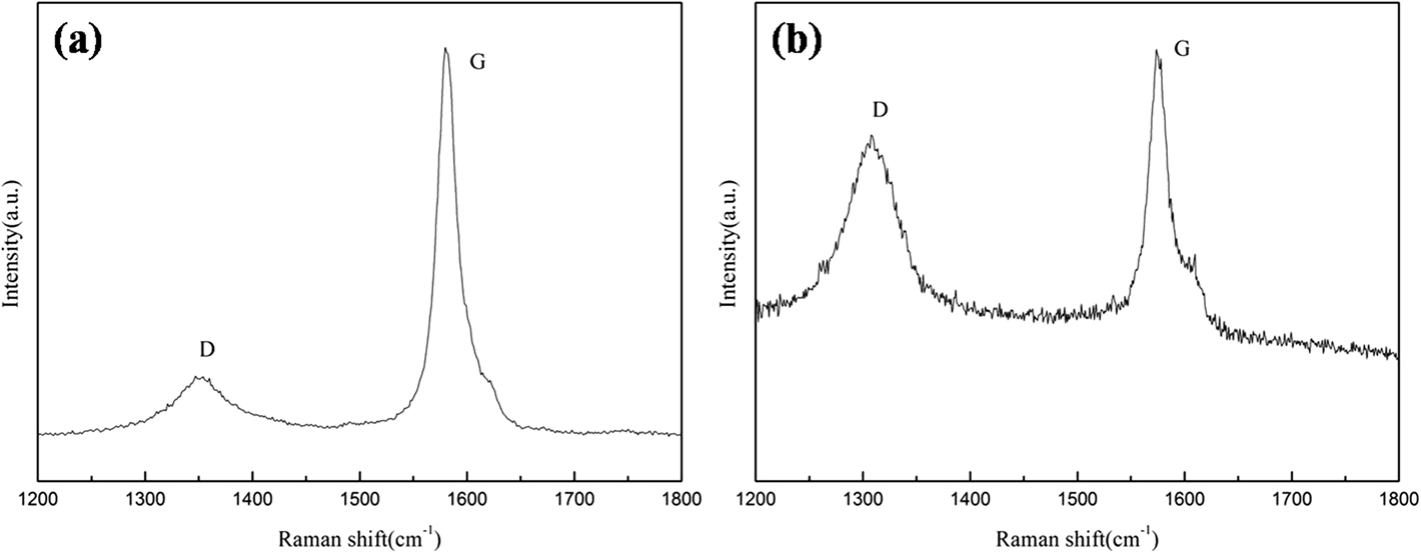
**Raman spectra of (a) f-GNPs and (b) SiO**_**2**_**/GNPs hybrid material.**

The FTIR spectra of f-GNPs, PAA-GNPs, siloxane-GNPs, and SiO_2_/GNPs hybrid material were presented in Figure 2. The peaks at 3,440 cm^−1^ (Figure 2a) which were attributed to stretching vibration of O-H groups could be observed clearly. The results indicated that GNPs had been functionalized successfully as designed. The peaks at 1,190 and 1,100 cm^−1^ (Figure 2b) were assigned to stretching vibration of C-O-C groups between GNPs and PAA, which indicated that PAA was grafted onto the surface of GNPs successfully. As showed in Figure 3c, the peaks at 1,556 and 3,300 cm^−1^ were attributed to bending vibration and stretching vibrating of N-H groups of amide, respectively. And the peak at 1,640 cm^−1^ (Figure 2c) was attributed to stretching vibration of C = O groups of amide. Meanwhile, the peaks at 1,121 and 1,045 cm^−1^ were attributed to stretching vibrating of Si-O and C-O groups of siloxane respectively. Also, the peak at 2,930 cm^−1^ was assigned to stretching vibration of C-H groups of alkyl groups. All these features confirmed that KH550 have linked with PAA-GNPs successfully. Figure 2d showed the spectrum of SiO_2_/GNPs hybrid material, compared with Figure 2c; it was clear that there appeared new stretching vibration peak of Si-O-Si groups at about 1,096 cm^−1^, and the peak at 796 cm^−1^ was attributed to the symmetric stretching of Si-O-Si groups as designed in Figure 1. All these data indicated that SiO_2_ fabricated on the surface of GNPs successfully.

### Raman spectra

Raman spectroscopy is a powerful and useful technique to investigate the ordered or disordered crystal structures and assessing defects of graphene-based materials. It is well known that the typical features of carbon materials in Raman spectra are the G band at 1,580 cm^−1^ deriving from the E_2g_ phonon of C sp^2^ atoms and D band at 1,350 cm^−1^ considered as a breathing mode of k-point photos of A_1g_ symmetry which is assigned to local defects and disorder mostly at the edges of f-GNP platelet [33,34].

Raman spectra of f-GNP and SiO_2_/GNPs hybrid material were shown in Figure 3. The D band at 1,352 cm^−1^ and G band at 1,580 cm^−1^ of f-GNP could be seen clearly in Figure 3a. While the D band at 1,308 cm^−1^ and G band at 1,575 cm^−1^ of f-GNPs/SiO_2_ hybrid materials could be seen clearly in Figure 2b. The shifting (from 1,352 to 1,308 cm^−1^) of D band was correlated with dramatic structural changes, associated with the changes of chemical bond between f-GNPs and SiO_2_. According to our analysis, the *I*_D_/*I*_G_ of f-GNPs and SiO_2_/GNPs hybrid material was 0.814 and 1.145, respectively (Table 3). The intensity ratio of the D and G bands (*I*_D_/*I*_G_) is a measure of the reduction degree, which consists with the sp^3^/sp^2^ carbon ratio, and the increasing in *I*_D_/*I*_G_ demonstrated that sp^3^ or disordered carbon atoms increased and carbon domains were destroyed [35,36]. The increased *I*_D_/*I*_G_ intensity ratio from 0.814 to 1.145 after chemical reaction could be attributed to covalent bond formation between f-GNPs and SiO_2_ which could generate a considerable number of defect sites in the graphene structure. Thus, the Raman data suggested that after chemical reacting the surface of f-GNPs nanosheets was disordering seriously.

**Table 3:** **Intensity ratio of the D and G bands (*I***_**D**_**/*I***_**G**_**)**

**Samples**	**D area**	**G area**	***I***_**D**_**/*I***_**G**_
f-GNPs	257,462	316,479	0.814
SiO_2_/GNPs	380,603	332,156	1.145

### Thermal gravimetric analysis

**Figure 4:**
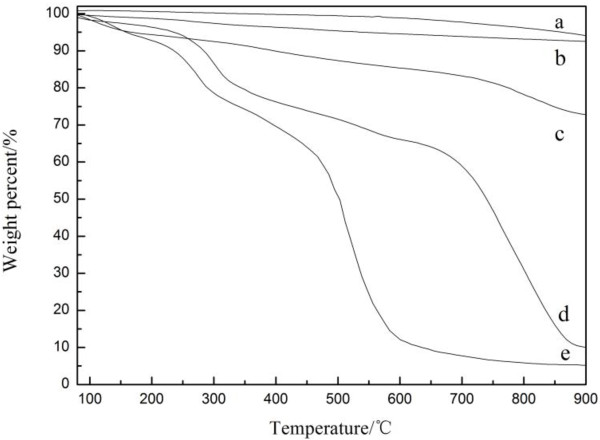
**TGA curve spectrum diagram.** (curve a) SiO_2_, (curve b) f-GNPs, (curve c) SiO_2_/GNPs hybrid material, (curve d) siloxane-GNPs, and (curve e) PAA-KH550.

Figure 4 presented the TGA curves for all the samples. As shown in Figure 3a, the raw SiO_2_ kept stable without significant weight loss until 900°C. The final weight-loss ratio of neat SiO_2_ particles was about 6.0%, which was caused by resolving of hydroxyl and carboxyl. Similarly, the f-GNPs (Figure 3b) kept stable without significant weight loss until 900°C, too. The final weight-loss ratio of f-GNPs was about 7.5%, which was caused by resolving of hydroxyl and carboxyl. SiO_2_/GNPs hybrid material (trace c) kept stable without significant weight loss until 700°C, and it had a slight weight reduction from 700°C to 900°C as shown in Figure 4. SiO_2_/GNPs hybrid material lost about 27% of its original weight in the end, which could be undoubtedly assigned to thermal decomposition of polymer. Thus, it suggested that the SiO_2_/GNPs hybrid material we have prepared possessed stable thermal stability. As shown in Figure 4d, there was a shape reduction of weight and two stages of weight loss for siloxane-GNPs could be identified, the first stage from 200°C to 350°C and the second stage from 600°C to 880°C. The first stage was associated to the resolving of hydroxyl and carboxyl on the surface of f-GNPs and removal of the H_2_O vapors of the sample; the major weight loss between 600°C and 880°C could be undoubtedly assigned to the decomposition of molecular chain of polymer. The final weight-loss ratio of siloxane-GNPs was about 90% in the end. PAA-KH550 polymer (trace e) lost about 95% of its original weight in the end, and two stages of weight loss for PAA-KH550 could be identified, the first stage from 200°C to 400°C was associated to the decomposition of the side groups of PAA-KH550 polymer. And the major weight loss in the second stage from 400°C to 650°C could be undoubtedly assigned to the decomposition of molecular chain of polymer. The final weight-loss ratio of PAA-KH550 polymer was about 95% in the end. Comparing the five traces, the weight fraction of PAA-KH550 polymer on siloxane-GNPs and that of SiO_2_ on SiO_2_/GNPs hybrid material could be estimated by following equations [19]:

(1) $$C\%=\left(1-X\right)*A\%+X*D\%$$

(2) $$B\%=\left(1-Y\right)*C\%+Y*E\%$$

where A%, B%, C%, D%, and E% were the weight loss percentages at a certain temperature of f-GNPs, SiO_2_/GNPs hybrid material, siloxane-GNPs, PAA-KH550, and SiO_2_, respectively. *X* and *Y* were denoted as the weight fraction of polymeric species on siloxane-GNPs and content of SiO_2_ on SiO_2_/GNPs hybrid material, respectively.

According to our calculation, At 900°C, the residual weight fraction of polymer on siloxane-GNPs was about 94.2% and that of SiO_2_ particles on hybrid materials was about 75.0%.

### Scanning electron microscopy

**Figure 5:**
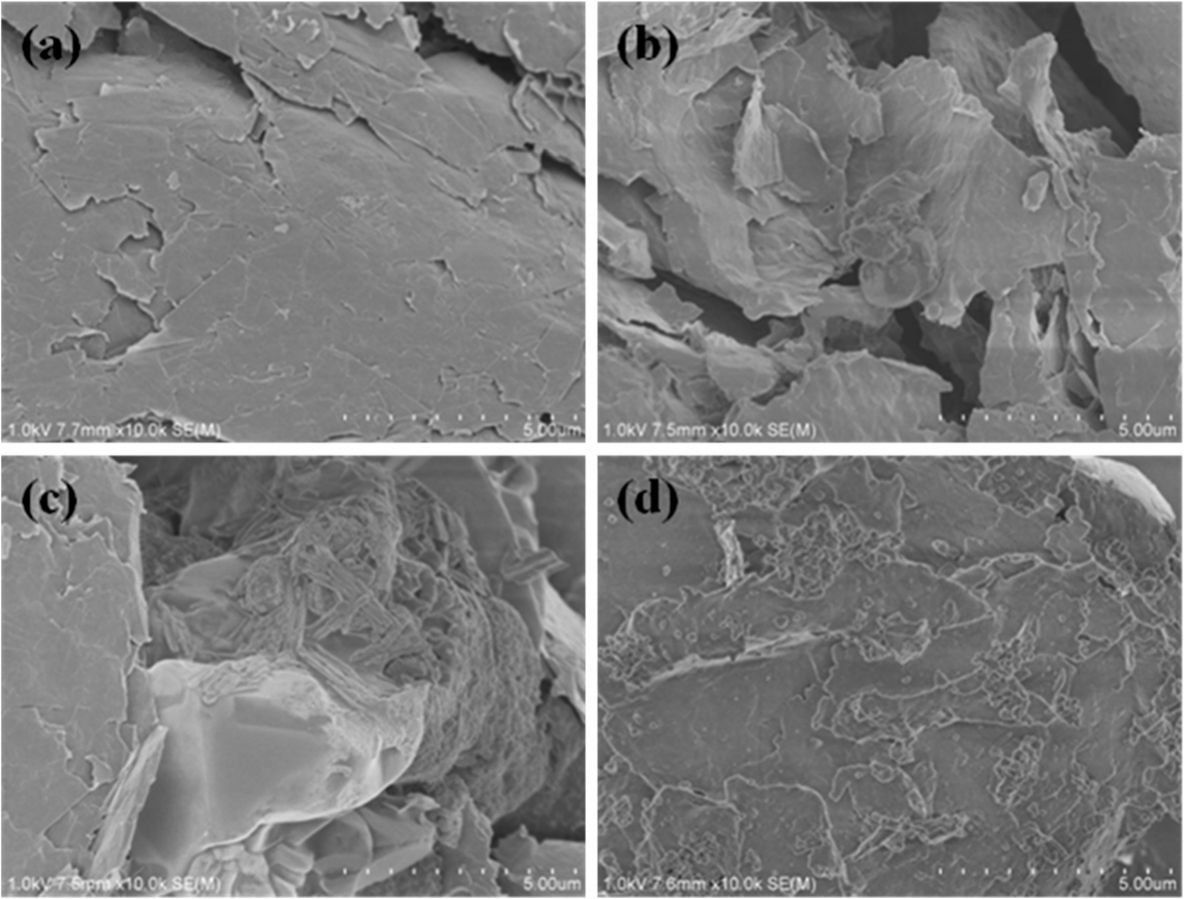
**SEM images of (a) f-GNPs, (b) PAA-GNPs, (c) siloxane-GNPs and (d) SiO**_**2**_**/GNPs.**

Figure 5 presented the SEM micrographs of the morphology of various GNPs samples. f-GNPs in lateral dimension were shown in Figure 5a, which were crumpled due to the transformation from a planar sp^2^-hybridized to a distorted sp^3^-hybridized geometry during the oxidation process. As shown in Figure 5b, after reacted with PAA, the sheet of GNPs appeared thicker in its thickness. Figure 5c showed micrographs of siloxane-GNPs. There appeared polymer on the surface of GNPs because of reacting with KH550. As showed in Figure 5d, SiO_2_ particles were adsorbed on surface of f-GNPs nanosheets. From all the images and analysis above, it was reasonable to believe that SiO_2_ particles have grown on the surface of GNPs successfully.

### Transmission electron microscopy

**Figure 6:**
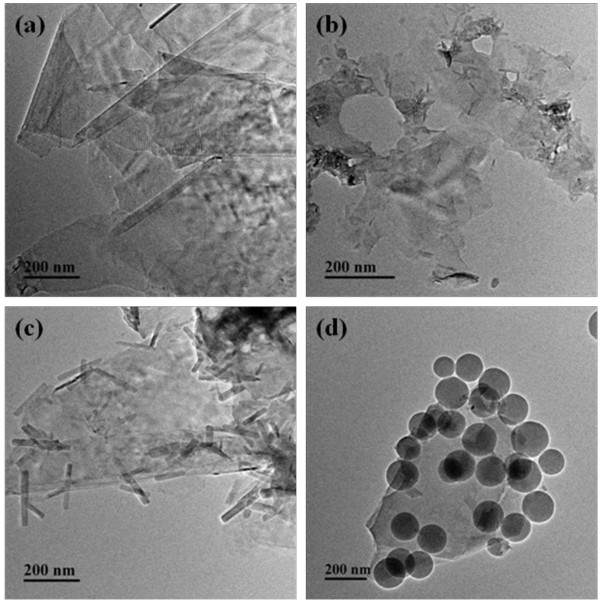
**TEM images of (a) f-GNPs, (b) PAA-GNPs, (c) siloxane-GNPs, and (d) SiO**_**2**_**/GNPs hybrid material.**

The typical morphologies of all samples were observed with TEM. As shown in Figure 6a, the surface of f-GNPs was relatively smooth and clean. After being functionalized with PAA, the surface of GNPs became blurred as shown in Figure 6b. After reacted with KH550, the functionalized GNPs (Figure 6c) became thickened and there appeared tubes on the surface of GNPs. The typical morphologies of SiO_2_/GNPs hybrid material were showed in Figure 6d. It was clear to discern that the SiO_2_ particles were hanged on the surface of f-GNPs. And the diameter of SiO_2_ varies from 100 to 200 nm. The TEM images were consistent with the result of the SEM, which confirmed that our route of preparing SiO_2_/GNPs hybrid material was reasonable.

**Figure 7:**
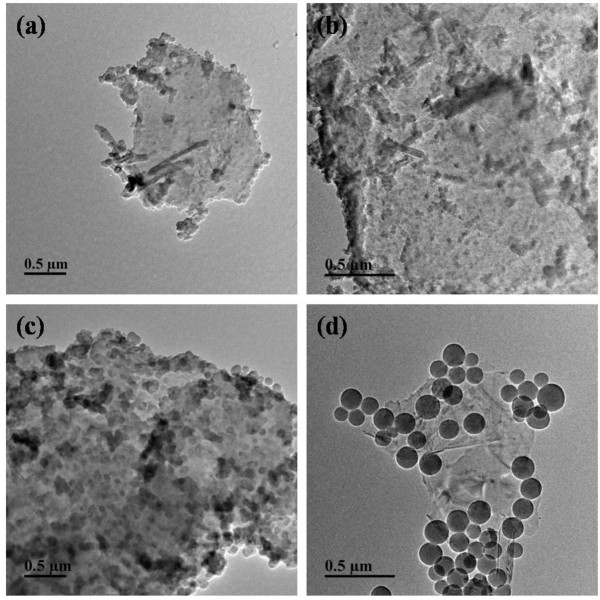
**TEM images of the growing process of SiO**_**2**_**/GNPs hybrid material with different times. (a)** 2 h, **(b)** 4 h, **(c)** 6 h, and **(d)** 8 h.

Figure 7 depicted the whole growth process of SiO_2_ particles on the surface of graphene with the ammonia of 1.2 g and TEOS of 0.6 g. When the reaction time was 2 h (Figure 7a), we can see that graphene became thicker and there appeared polymeric substance on the surface of graphene. As the reaction time reached 4 h (Figure 7b), SiO_2_ particles did not completely grow, but some little black points could be observed which were the miniatures of SiO_2_ particles. With the time growing, it could be seen that the surface of graphene were covered with SiO_2_ particles when the reaction time was 6 h (Figure 7c); SiO_2_ particles became larger than that of Figure 7b, but had not completely grown to round shape. Figure 7d showed that after 8-h growing, SiO_2_ particles had grown fully, and the average size of SiO_2_ particles was 140 nm.

### Analysis of orthogonal experiment

According to the matrix, nine experiments were carried out and the average size of SiO_2_ particles was shown in Table 2. This table showed that the range of the size of SiO_2_ particles varies from 50 to 280 nm; these data were taken as the original data and used in the range analysis. The mean values of Ij/kj, IIj/kj, and IIIj/kj for different factors at different levels in the range analysis were shown in Table 4. For each factor, a higher mean value indicates that the level has a larger effect on the size of SiO_2_ particles. And the range value indicates the significance of the factor's effect, and a larger range means the factor has a bigger impact on the size of SiO_2_ particles. Therefore, according to Table 4, compared with the range values of different factors, the factors' level of significance are as follows: ammonia (103.4) > TEOS (86.7) > reaction time (43.3). The range value of ammonia is the largest, which means that the quality of ammonia had the most important impact on the size of SiO_2_ particles.

**Table 4:** Analysis of range of each other

**Column no.**	***j*** **= 1**	**2**	**3**
**Factors**	**TEOS**	**NH**_**3**_^**.**^**H**_**2**_**O**	**Time**
Ij	I1 = 310	I2 = 280	I3 = 380
IIj	II1 = 510	II2 = 520	II3 = 500
IIIj	III1 = 570	III2 = 590	III3 = 510
kj	k1 = 3	k2 = 3	k3 = 3
Ij/kj	103.3	93.3	126.7
IIj/kj	170	173.3	166.7
IIIj/kj	190	196.7	170
Range	86.7	103.4	43.3

According to our analysis, the amount of ammonia affects the size of SiO_2_ particles most. With the increasing of the amount of ammonia from 0.6 to 1.8 g, the size of SiO_2_ particles increases continuously. The joining of ammonia can significantly contribute to the occurrence of hydrolysis and polycondensation reaction of TEOS. When adding NH_3_^.^H_2_O to the solution, the OH anion made the silicon atoms negatively charged. As a result, Si-O bond weakened and eventually cracked. The products of hydrolysis reaction such as Si-OH and Si-OR dehydration or dealcoholation in the next polycondensation processing form Si-O-Si chain. Si-O-Si chains cross-linked continuously with each other to fabricate SiO_2_ particles finally. The hydrolysis rate will increase with the growing amount of ammonia, so the size of SiO_2_ particles also becomes larger.

With the increasing of the amount of TEOS from 0.3 to 0.9 g, the size of SiO_2_ particles also increases continuously. From the viewpoint of chemical equilibrium, the increasing of the content of TEOS contributes to the hydrolysis reaction to form SiO_2_ particles. However, the influence of TEOS is not as significant as ammonia.

The reaction time also had impact on the results. The size of SiO_2_ particles grew with the increasing of the reaction time from 4 to 8 h. With the time increasing, the cross-linking between Si-O-Si chains strengthened, and the size of SiO_2_ particles became larger and larger.

According to the above analysis, the controllability of the particle sizes was realized and in a certain range, the quantity of ammonia, the quantity of TEOS and the reaction time all had positive effect on the growing of SiO_2_ particles.

## Conclusion

In this work, SiO_2_/GNPs hybrid material had been successfully achieved by a facile and controllable method as designed. In this process, firstly, PAA was grafted to the surface of f-GNPs for providing reaction pots, and then KH550 reacted with abovementioned product PAA-GNPs to obtain siloxane-GNPs. Finally, the SiO_2_/GNPs hybrid material is produced through introducing siloxane-GNPs into a solution of tetraethyl orthosilicate, ammonia, and ethanol for hours' reaction. The new characteristic band from FTIR indicated that those chemical reactions had been occurred as designed, and the results from SEM and TEM indicated that SiO_2_ nanoparticles were grown on the surface of f-GNPs successfully. Raman spectroscopy proved that after chemical drafting disordered, carbon atoms increased and carbon domains were destroyed. TGA traces suggested the residual weight fraction of polymer on siloxane-GNPs was about 94.2% and that of SiO_2_ particles on hybrid materials was about 75.0% finally and the SiO_2_/GNPs hybrid material we have prepared had stable thermal stability. Therefore, it was a feasible and reliable route to produce SiO_2_/GNPs hybrid material. Through orthogonal experiments, we also got the result that the controllability of the particle sizes was realized and the amount of ammonia had the most important impact on the size of SiO_2_ particles compared with quantity of TEOS and the reaction time. The next target of our study is to do research on the application of the hybrid material, to prepare epoxy resin composites with hybrid material, and study the influence of the SiO_2_ particles' size to strengthen epoxy resin composites.

## Abbreviations

APTES: 3-aminopropyltriethoxysilane; f-GNPs: functionalized graphene nanoplatelets; FTIR: Fourier transform infrared spectra; PAA: polyacrylic acid; SEM: scanning electron microscopy; SiO2: silica nanoparticles; SiO2/GNPs: SiO_2_/graphene nanoplatelets; TEM: transmission electron microscopy; TGA: thermal gravimetric analysis.

## Competing interests

The authors declare that they have no competing interests.

## Authors’ contributions

KY, KQ, HC, XL, and JS gave the guidance, JL did the experiments, analyzed the data, and gave the final approval of the version of the manuscript to be published. All authors read and approved the final manuscript.

## References

[B1] Novoselov KS Geim AK Morozov SV Jiang D Zhang Y Dubonos SV Grigorieva IV Firsov AA Electric field effect in atomically thin carbon films Science 2004 9 666 669 10.1126/science.1102896 15499015

[B2] Castro NAH Guinea F Peres NMR Novoselov KS Geim AK The electronic properties of graphene Rev Mod Phys 2009 9 109 162 10.1103/RevModPhys.81.109

[B3] Rao CNR Sood AK Subrahmanyam KS Govindaraj A Graphene: the new two-dimensional nanomaterial Angew Chem Int Ed 2009 9 7752 7777 10.1002/anie.200901678 19784976

[B4] Geim AK Novoselov KS The rise of graphene Nat Mater 2007 9 183 191 10.1038/nmat1849 17330084

[B5] Stankovich S Dikin DA Dommett GHB Kohlhaas KM Zimney EJ Stach EA Piner R Nguyen ST Ruoff RS Graphene-based composite materials Nature 2006 9 282 286 10.1038/nature04969 16855586

[B6] Xu C Wang X Zhu J Graphene-metal partical nanocomposites J Phys Chem C 2008 9 19841 19845 10.1021/jp807989b

[B7] Wen YY Ding HM Shan YK Preparation and visible light photocatalytic activity of Ag/TiO_2_/graphene nanocomposite Nanoscale 2011 9 4411 4417 10.1039/c1nr10604j 21909581

[B8] Sreeprasad TS Maliyekkal SM Lisha KP Pradeep T Reduced graphene oxide-metal/metal oxide composites: facial synthesis and application in water purification J Hazard Mater 2011 9 921 931 10.1016/j.jhazmat.2010.11.100 21168962

[B9] Muszynski R Seger B Kamat PV Decorating graphene sheets with gold nanoparticles Phys Chem C 2008 9 5263 5266 10.1021/jp800977b

[B10] Seema H Kemp KC Chandra V Kim KS Graphene-SnO_2_ composites for highly efficient photocatalytic degradation of methylene blue under sunlight Nanotechnology 2012 9 355705 355712 10.1088/0957-4484/23/35/355705 22894878

[B11] Hao LY Song HJ Zhang LC Wan XY Tang YR Lv Y SiO2/graphene composite for highly selective adsorption of Pb(II) ion J Colloid Interface Sci 2012 9 381 387 10.1016/j.jcis.2011.12.023 22218342

[B12] Vaisman L Marom G Wagner HD Dispersions of surface-modified carbon nanotubes in water-soluble and water-insoluble polymers Adv Funct Mater 2006 9 357 363 10.1002/adfm.200500142

[B13] Breuer O Sundararaj U Big returns from small fibers: a review of polymer/carbon nanotube composites Polym Compos 2004 9 630 645 10.1002/pc.20058

[B14] Xu LQ Liu YL Neoh KG Kang ET Fu GD Reduction of graphene oxide by aniline with its concomitant oxidative polymerization Macromol Rapid Commun 2011 9 684 688 10.1002/marc.201000765 21480428

[B15] Williams G Seger B Kamat PV TiO_2_-Graphene nanocomposites. UV-assisted photocatalytic reduction of graphene oxide ACS Nano 2008 9 7 1487 1491 10.1021/nn800251f 19206319

[B16] Shen JF Hu YZ Shi M Li N Ma HW Ye MX One step synthesis of graphene oxide-magnetic nanoparticle composite J Phys Chem C 2010 9 3 1498 1503 10.1021/jp910013f

[B17] Si YC Samulski ET Exfoliated graphene separated by platinum nanoparticles Chem Mater 2008 9 6792 6797 10.1021/cm801356a

[B18] Kim H Abdala AA Macosko CW Graphene/polymer nanocomposites Macromolecules 2010 9 6515 6530 10.1021/ma100572e

[B19] Zhou HF Zhang C Li HQ Du ZJ Fabrication of silica nanoparticles on the surface of functionalized multi-walled carbon nanotubes Carbon 2011 9 126 132 10.1016/j.carbon.2010.08.051

[B20] Li X Liu Y Fu L Cao L Wei D Wang Y Efficient synthesis of carbon nanotubes–nanoparticle hybrids Adv Funct Mater 2006 9 18 2431 2437 10.1002/adfm.200600339

[B21] Zhang Y Shen Y Han D Wang Z Song J Niu L Reinforcement of silica with single-walled carbon nanotubes through covalent functionalization J Mater Chem 2006 9 47 4592 4597 10.1039/b612317a

[B22] Bottini M Magrini A Marcia I Bergamaschi A Mustelin T Non-destructive decoration of full-length multi-walled carbon nanotubes with variable amounts of silica gel nanoparticles Carbon 2006 9 1301 1303 10.1016/j.carbon.2006.01.003

[B23] Song H Zhang L He C Qu Y Tian Y Lv Y Graphene sheets decorated with SnO_2_ nanoparticles: in situ synthesis and highly efficient materials for cataluminescence gas sensors J Mater Chem 2011 9 5972 5977 10.1039/c0jm04331a

[B24] Zhou X Shi TJ One-pot hydrothermal synthesis of a mesoporous SiO2-graphene hybrid with tunable surface area and pore size Appl Surf Sci 2012 9 566 573

[B25] Zhang K Dwivedi V Chi CYJ Wu JS Graphene oxide/ferric hydroxide composites for efficient arsenate remol from drinking water Hazard Mater 2010 9 162 168 10.1016/j.jhazmat.2010.06.010 20580161

[B26] Chandra V Park J Chun Y Lee JW Hwang IC Kim KS Water-dispersible magnetite-reduced graphene oxide composites for arsenic removal ACS Nano 2010 9 7 3979 3986 10.1021/nn1008897 20552997

[B27] Xu C Wang X Zhu J Yang XJ Lu L Deposition of Co_3_O_4_ nanoparticles onto exfoliated graphite oxide sheets J Mater Chem 2008 9 5625 5629 10.1039/b809712g

[B28] Agrawal S Kumar A Frederick MJ Ramanath G Hybrid microstructures from aligned carbon nanotubes and silica particles Small 2005 9 823 826 10.1002/smll.200500023 17193532

[B29] Bottini M Tautz L Huynh H Monosov E Bottini N Dawson MI Bellucci S Mustelin T Covalent decoration of multi-walled carbon nanotubes with silica nanoparticles Chem Commun 2005 9 6 758 760 10.1039/b412876a 15685328

[B30] Lu WB Luo YL Chang GH Sun XP Synthesis of functional SiO_2_-coated graphene oxide nanosheets decorated with Ag nanoparticles for H_2_O_2_ and glucose detection Biosens Bioelectron 2011 9 4791 4797 10.1016/j.bios.2011.06.008 21733668

[B31] Hu QW Fang PF Dai YQ Effect of the reactant concentration on the particle sizes of monodispersed silica nanoparticles Bull Chin Ceramic Soc 2012 9 5 1218 1222

[B32] Wu X Leung DYC Optimization of biodiesel production from camelina oil using orthogonal experiment Appl Energy 2011 9 11 3615 3624 10.1016/j.apenergy.2011.04.041

[B33] Akhavan O The effect of heat treatment on formation of graphene thin films from graphene oxide nanosheets Carbon 2010 9 509 519 10.1016/j.carbon.2009.09.069

[B34] Kudin KN Ozbas B Schniepp HC Prud’homme RK Aksay IA Car B Raman spectra of graphite oxide and functionalized graphene sheets Nano Lett 2008 9 36 41 10.1021/nl071822y 18154315

[B35] Mohanty N Nagaraja A Armesto J Berry V High-throughput, ultrafast synthesis of solution-dispersed graphene via a facile hydride chemistry Small 2010 9 226 231 10.1002/smll.200901505 19943253

[B36] Gengler RYN Veligura A Enotiadis A Diamanti EK Gournis D Jozsa C Wees BJV Rudolf P Large-yield preparation of high-electronic-quality graphene by a Langmuir–Schaefer approach Small 2010 9 35 39 10.1002/smll.200901120 19937610

